# GACEM: Genetic Algorithm Based Classifier Ensemble in a Multi-sensor System

**DOI:** 10.3390/s8106203

**Published:** 2008-10-01

**Authors:** Rongwu Xu, Lin He

**Affiliations:** Institute of Noise & Vibration, Naval University of Engineering, Wuhan 430033, P. R. China; E-mails: r.xu@ieee.org, helin202@public.wh.hb.cn

**Keywords:** Genetic algorithm, classifier ensemble, multi-sensor system, optimization, fusion

## Abstract

Multi-sensor systems (MSS) have been increasingly applied in pattern classification while searching for the optimal classification framework is still an open problem. The development of the classifier ensemble seems to provide a promising solution. The classifier ensemble is a learning paradigm where many classifiers are jointly used to solve a problem, which has been proven an effective method for enhancing the classification ability. In this paper, by introducing the concept of Meta-feature (MF) and Trans-function (TF) for describing the relationship between the nature and the measurement of the observed phenomenon, classification in a multi-sensor system can be unified in the classifier ensemble framework. Then an approach called Genetic Algorithm based Classifier Ensemble in Multi-sensor system (GACEM) is presented, where a genetic algorithm is utilized for optimization of both the selection of features subset and the decision combination simultaneously. GACEM trains a number of classifiers based on different combinations of feature vectors at first and then selects the classifiers whose weight is higher than the pre-set threshold to make up the ensemble. An empirical study shows that, compared with the conventional feature-level voting and decision-level voting, not only can GACEM achieve better and more robust performance, but also simplify the system markedly.

## 1. Introduction

Classification is one of the most important purposes of multi-sensor systems (e.g., target recognition [1, 2], personal identity verification [3], landmine detection [4]). It is well known that data available from multiple sources underlying the same phenomenon may contain complementary information. Intuitively, if such information from multiple sources can be appropriately combined, the performance of a classification system could be improved. A classification system, capable of combining information from multiple sources or from multiple feature sets, is said to be capable of performing data fusion. Usually there are two conventional approaches to deal with this, i.e., feature-level fusion and decision-level fusion [2, 5-7]. In feature-level fusion, features are extracted from multiple sensor observations, and combined into a single concatenated feature vector which is input to a classifier such as neural networks, decision trees, etc. Decision-level fusion involves the fusion of sensor information, after each sensor has made a preliminary solution of the classification task [8]. There have been some qualitative suggestions about how to choose the fusion strategy: Brooks [6] supposed that feature-level fusion would be a superior choice if the information represented by the data was correlated, while decision-level fusion would be a better choice if the data was uncorrelated. Additionally, in [9] it was demonstrated that decision-level fusion worked well when the data was fault-free, but its performance degraded faster than feature-level fusion when measurement error was introduced to the system. However, most of these conclusions are from empirical research and neither data fusion nor decision fusion can be proven to be the optimal fusion technique for all events, so the search for the optimal fusion framework in multi-sensor systems is still an open problem.

In the last decade, quite a lot of papers have proposed a classifier ensemble for designing high performance pattern classification systems [10, 11]. A classifier ensemble is also known under different names in the literature: combing classifiers, committees of learners, mixtures of experts, classifier fusion, multiple classifier systems, etc [12]. It has been proven that in the long run, the combined decision is supposed to be better (more accurate, more reliable) than the classification decision of the best individual classifier [13]. Generally, the research on classifier ensembles involves two main phases: the design of the ensemble process and the design of the combination function. Although this formulation of the design problem leads one to think that effective design should address both phases, most of the design methods described in the literature focus on only one of them [10, 14]. For the multi-sensor system, as we know, there is not so much research focused on the application of classifier ensembles. Ref. [15] argued that application of classifier ensembles in the decision-level fusion could be helpful for *moderation* to compensate for sampling problems where moderation can be regarded as replacing any fusion parameter's value with its mathematical expectation. But the results could be better convinced if there is a large-scale empirical study for proof and it is almost impossible to moderate sophisticated classifier, such as neural networks, because of the high variability of excessive parameters. Another approach proposed in [16] by Polikar *et al.* is generating an ensemble of classifiers using data from each source, and combining these classifiers using a weighted voting procedure. The weights are determined based on the individual classifier's training performance as well as the observed or predicted reliability of each data source. In essence, the approach is derived from AdaBoost [17] which involves subsampling the training examples [18]. We have also shown an analogous application of the Bagging algorithm [19] in mechanical noise source identification [20]. Moreover, Roli *et al.* presented an application of classifier fusion for multi-sensor image recognition [21]. The common feature is that Refs. [16, 20, 21] mostly focused on the decision level. As shown in later sections (see Section 2.3), we believe that these approaches could be synergistic with the new method proposed in this article.

In this paper, an approach named Genetic Algorithm based Classifier Ensemble in Multi-sensor system (GACEM) is proposed. By introducing the concept of Meta-feature (MF) and Trans-function (TF), the fusion problem can be unified in the classifier ensemble framework and then it has been shown that either the feature-level fusion and or the decision-level fusion is just a special case of our framework. After that, different from the previous application of GA [22, 23], an *ad hoc* chromosome coding strategy in GACEM is presented for the selection of feature subset and the optimization of decision combination simultaneously. Correspondingly, some genetic operators such as crossover and mutation operators are modified to take into account a binary and real-coded chromosome template. By doing so, the final classifier ensemble framework is obtained after evolution. Finally, an experiment of classification of 35 kinds of different sound sources is designed and the results prove the effectiveness of GACEM.

The paper is organized as follows. In the next section we analyze the feasibility of application of classifier ensemble in multi-sensor system. The technical detail of GACEM is discussed in Section 3. Section 4 provides and analyzes the experimental results of sound source classification. Finally, conclusions and some potential further research directions are presented in Section 5.

## 2. Problem Formulation and Analysis

### 2.1 Problem formulation

Consider a classification problem where a test pattern (whch may be an event, a physical phenomenon, etc.) is to be assigned to a class label *S* (*S*∈{*s*_1_, *s*_2_,…,*s_L_*}, *L* is the number of possible classes). And measuring the test pattern is carried out by means of *M* sensors. Here the sensors may be heterogeneous or homogeneous. Let us assume that the observations on the test pattern from the *i* -th sensor is represented by feature vector *R_i_* (*i* = 1,…*M*). Without the loss of generality, *R_i_* (*i* = 1,…*M*) is assumed to be a row feature vector. Now the goal is to find the most appropriate mapping from the observation set {*R*_1_,…*R_M_*} to the pattern class label *S*.

**Figure 1.**
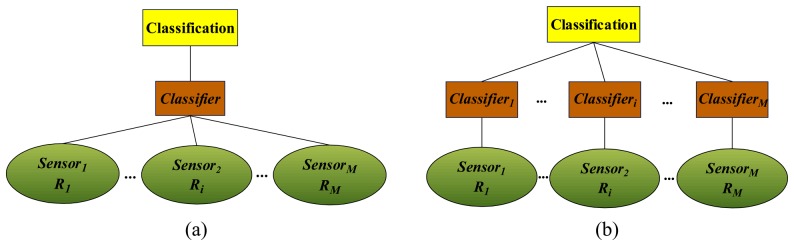
Demonstration of (a) feature-level fusion and (b) decision-level fusion.

The conventional avenues for the problem are shown in Figure 1, i.e., (a) feature-level fusion and (b) decision-level fusion. As shown in Figure 1(a), the features for training can be expressed as [*R*_1_ċ*R_M_*] and the single classifier is trained based on the features from all sensors, while in Figure 1(b), the *i* -th classifier is trained only on the feature vector *R_i_* and then all the classification results are combined to form a comprehensive decision through a given strategy such as voting or averaging.

### 2.2 Definition of Meta-feature and Trans-function

As mentioned above, *R_i_* can be considered a quantitative estimation of the test pattern's characters using the *i*-th sensor. Intuitively, it is believed that different sensors probably give different measurements due to the factors of sensor type, position, sensitivity, etc. But it is worth noting that they are describing the *same* test pattern after all. So there must be some kind of inherent relationship among them. Here we call *R_0_ Meta-feature* (MF) which is defined as the intrinsic and natural expression of the test pattern's characters, which is probably a priori in most situations. Suppose there is a functional relationship *T_i_* between *R_0_* and *R_i_*, i.e., *R_i_*=*T_i_*(*R*_0_). Then we define *T_i_* as the *Transfunction* (TF) from *R*_0_ to *R_i_*, ∀*i*∈ [1,*M*]. Specially, if *R_i_* is the same as *R*_0_, then the TF is invariant, i.e., *R_i_*=*T_i_*(*R*_0_) = *R*_0_.

The concepts of MF and TF are the theoretical basis of applying classifier ensemble methods in multi-sensor systems. Unfortunately, in many situations, the concept of MF and TF may be hard to substantialize and understand, so they are of less use for calculation than theoretical deduction. But under certain conditions, they do have exact physical meaning. For example, in the sound measurement system (see Section 4.1), if we use the power spectrum as the feature vector, then *R*_0_ is the power spectrum at the excitation point (sound source position) and *T_i_* is in fact equivalent to the square of magnitude of the frequency response function (FRF) between the excitation point and the *i*-th response point (sensor position). And given a precise system model (e.g., the finite element model built in ANSYS), all the information mentioned above can be calculated.

### 2.3 Classifier ensembles in multi-sensor systems

Using MF and TF, the observation set {*R*_1_,…*R_M_*} can be rewritten as {*T*_1_(*R*_0_),…,*T_M_*(*R*_0_)}. And then the classification problem can be modified as: how to find the most appropriate function *H*(*T*_1_(*R*_0_),…,*T_M_*(*R*_0_)) which is the mapping from the observation set {*T*_1_(*R*_0_),…,*T_M_*(*R*_0_)} to the pattern class label *S*. Without the loss of generality, define a single-variable function *H_0_* to replace the multi-variable function *H*, i.e., *H*_0_(*R*_0_) = *H*(*T*_1_(*R*_0_),…,*T_M_*(*R*_0_)). Here it is obvious that the classification problem in Multi-sensor System (MSS) is in essence identical with the commonly used concept of pattern classification in non-MSS. That is to say, any technique proven to be effective in pattern classification is also believed to be theoretically effective in pattern classification in MSS.

**Figure 2.**
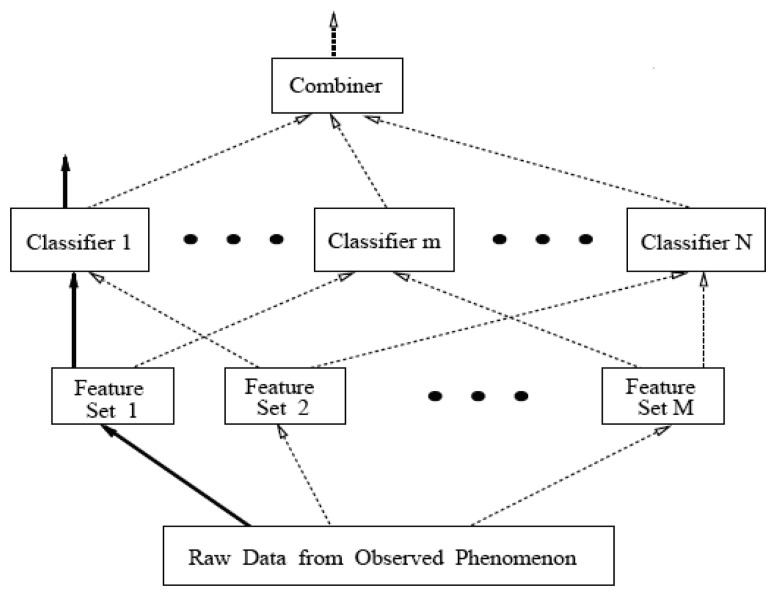
General framework of classifier ensemble.

Many researchers have shown that the classifier ensemble is a very promising way to improve classification performance [10, 11, 21] and a typical demonstration figure of a classifier ensemble can be found in [24]. As shown in Figure 2, several feature sets are generated from the raw data from an observed phenomenon and then a number of classifiers can be obtained by training from versatile combination of different feature sets. It is notable that the numbers of feature sets (*M*) and classifiers (*N*) may be unequal. Finally, on the base of classification of each classifier, the final classification result can be given through some kind of fusion rules, such as majority voting [25], plurality voting [26], weighted averaging [27].

**Figure 3.**
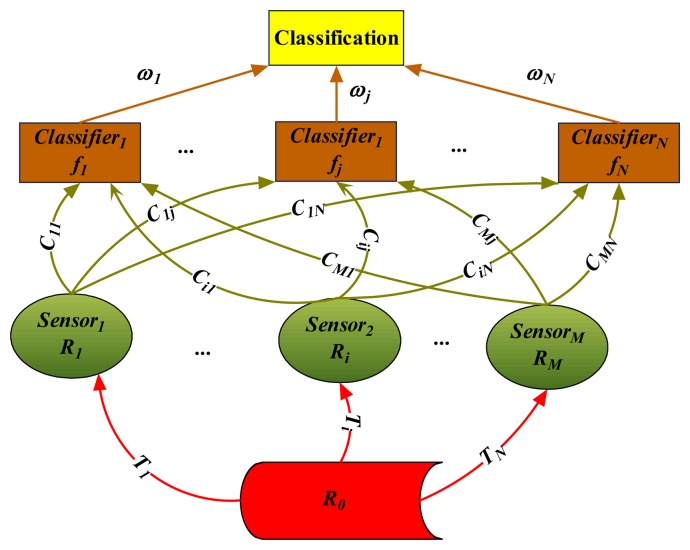
Framework of classifier ensemble in multi-sensor system.

Analogously, in MSS, the feature vector *R_i_* (∀*i*∈ [1,*M*]) is also generated from the MF *R*_0_ describing the observed phenomenon. What's more, the combination of feature vectors from different sensors will lead to versatile classifiers. As shown in Figure 3, the red line *T_i_* means the TF from *R*_0_ to feature vector *R_i_* (∀*i*∈ [1,*M*]). The green line *C_ij_* (∀*i*∈ [1,*M*], *j*∈ [1,*N*]) are binary (0-1) parameters representing whether the feature vector *R_i_* contributes to the training of the *j*-th classifier *f_j_*, i.e., *C_ij_* = 1 means positive and 0 negative. Besides, the importance of the *j*-th classifier can be indicated by *ω_j_*. Besides, it is very important to understand that the generated classifier *f_j_* may be a *sub*-classifier ensemble system by performing such operations like Bagging or Boost as mentioned in [16] or [20]. This, however, is not the focus here. Further studies will be summarized in our next study.

In particular, two special cases are given:

(1) $$\{\begin{array}{ll} C_{i1}=1, & \forall i\in [1,M]\\ C_{ij}=0, & \forall i\in [1,M],j\in [2,N]\\ w_{1}\neq 0 & \end{array}$$

and

(2) $$\{\begin{array}{ll} C_{ii}=1, & \forall i\in [1,M]\\ C_{ij}=0, & \forall i\in [1,M],j\in [1,M],i\neq j\\ M=N & \end{array}$$

Obviously, (1) is in accordance with the feature-level fusion [see Figure 1(a)] and (2) is in accordance with the decision-level fusion [see Figure 1(b)]. Next, given a pool of *N* classifiers, there are a number of possible combining strategies to follow. But it is usually not clear which one may be the optimal for a particular problem. The simplest idea is to enumerate all possible solutions, i.e., assessing the classification accuracy on a validation set with all possible solutions and then choosing one exhibiting the best performance [10]. But the burden of exponential complexity of such search limits its practical applicability for larger systems. For example, If *M* = *N* = 5, the number of possible combination of feature will be $\overset{N}{\underset{j=1}{\text{∏}}}(\overset{M}{\underset{i=0}{\text{∑}}}C_{M}^{i})-1=2^{MN}-1\approx 3.36\times 10^{7}$. Considering there would be hundreds of sensors in large-scale MSS in engineering, the exhausted search is obviously unpractical for application. So we need more feasible search algorithm.

## 3. GACEM: Genetic Algorithm based Classifier Ensemble in a Multi-sensor System

In essence, searching for the optimal classifier ensemble framework in MSS belongs to the ‘optimization-centered’ problem while traditional optimization techniques often fail to meet the demands and challenges of highly dynamic and volatile information flow [28]. In the prevailing optimization approaches, the genetic algorithm (GA) provides a valuable alternative to traditional methods due to its inherent parallel nature and its ability of global optimization.

### 3.1 A brief introduction of GA

A genetic algorithm is a search algorithm based on the mechanics of natural selection and natural genetics. It efficiently utilizes historical information to obtain new search points with expected enhanced performance. In every generation, a new set of artificial individuals is created, using the information from the best of the old generation. Genetic algorithm combines the survival of the fittest from the old population with a randomized information exchange that helps to form new individuals with higher fitness. There are three basic genetic algorithm operators: selection, crossover, and mutation. Those operators combined with the proper fitness function definition constitute the main body of genetic algorithms [29]. GA has been used in various pattern recognition problems, such as image registration, semantic scene interpretation, and feature selection [28].

In summary, the GA search process typically comprises of the following steps:

Step 1. Randomly generate initial population of chromosomes.
Step 2. Evaluate fitness (objective function) of each chromosome.
Step 3. Are the termination criteria met? If YES, go to step 7. If NO, go to step 4.
Step 4. Generate new population by selecting pairs for mating, recombination using crossover and mutation.
Step 5. Evaluate fitness (objective function) of each new chromosome.
Step 6. Identify the fittest individual in the population. Go to step 3.
Step 7. End.

### 3.2 Detail of GACEM

In this section we present an approach, i.e. GACEM, to find the optimal classifier ensemble in MSS. As mentioned above, the purpose of GACEM is optimization for design of both the ensemble process and the combination function.

#### 3.2.1 Chromosome coding strategy

A customized coding strategy has been developed for our task. Given *M* sensors and *N* classifiers, the length of chromosome is (*MN* + *N*). The first part of chromosome has *MN* gene positions representing the binary value of *C_ij_* (∀*i*∈ [1,*M*], *j*∈ [1,*N*]) (We call it *b-Part*). And the second part contains *N* positions corresponding to the different decisions weights of *N* classifiers respectively (We call it *r-Part*). It is worth noting that they are real-value coded and a normalization step is to be performed, i.e., $w_{i}^{\prime }=\frac{w_{i}}{\overset{N}{\underset{i=1}{\text{∑}}}w_{i}}(\forall i\in [1,N])$, to keep sum of the weights as one.

**Figure 4.**
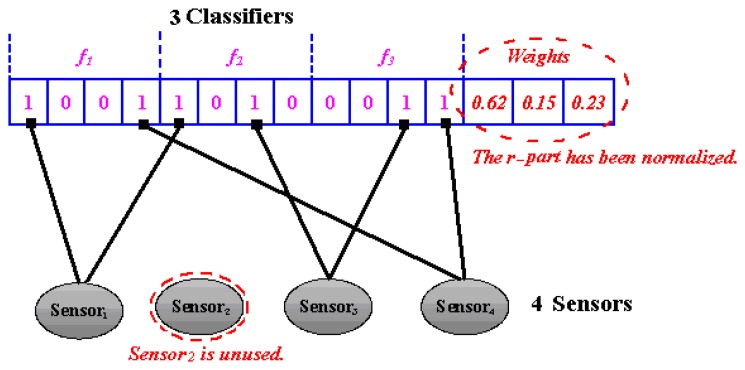
A possible chromosome when *M* = 4 and *N* = 3.

For example, if adopting weighted averaging as decision combination function, when *M* = 4 and *N* = 3, a possible chromosome coding is shown in Figure 4.

#### 3.2.2 Fitness function

Although there have been some studies on how to evaluate the performance of classifier ensembles and various measures have been proposed for the purpose [12], we don't think those heuristic statistical parameters are surely to be superior to directly choosing the classification accuracy as the criterion for evaluation. And it is believed that choosing an additional validation set other than the training set for evaluation will moderate the risk of overfitting [30]. So the classification performance on an evaluation sample set is adopted as the fitness function in GACEM.

#### 3.2.3 Selection operators

We choose the roulette selection in GACEM. The standard roulette selection chooses parents by simulating a roulette wheel, in which the area of the section of the wheel corresponding to an individual chromosome is proportional to its fitness performance.

#### 3.2.4 Crossover operator

Since there are both binary and real value codes in the chromosome, we need a hybrid crossover operator. For the *b-Part*, the scattered crossover function is adopted, which creates a random binary vector and selects the genes where the vector is a *1* from the first parent, and the genes where the vector is a *0* from the second parent, and combines the genes to form the child. While for the *r-Part*, we use the intermediate crossover function, which creates children by taking a weighted average of the parents. For example, if *p*_1_ and *p*_2_ are the parents: *p*_1_= < 0 0 1 0 1 1 ‖ 0.3 0.7 >, *p*_2_ = < 1 0 1 0 1 0 ‖ 0.4 0.6 >, the binary vector is [1 1 0 0 1 0] and the random ratio is 0.2. Then the children are: *c*_1_= < 0 0 1 0 1 0 ‖ 0.38 0.62 >, *c*_2_ = < 1 0 1 0 1 1 ‖ 0.32 0.68 >.

#### 3.2.5 Mutation operator

Mutation is also designed to be processed for different parts. For the *b-Part*, a random gene is chosen and the value *μ* is substituted by *NOT*(*μ*). While for the *r-Part*, another gene is chosen randomly and the value *μ* is replaced by a new random number between [0,1].

#### 3.2.6 Stopping criteria

There are two termination conditions in GACEM. Either the maximum number of iterations over the terminal number *I*_max_ of generations or the best fitness value beyond the value of fitness limit *L_fit_*, the algorithm will stop.

### 3.3 Flowchart

Now we have introduced most of the details of GACEM, but there is still another three important prerequisites before performing the algorithm: (1) choosing the basic classifier, (2) determination of *N* and (3) choosing the decision combination function. For (1), first it is notable that GACEM is classifier-independent, i.e., any classifier, such as a neural network (NN) or a decision tree (DT), could in theory be applied as basic classifier for the ensemble, but considering the fact that GA is inherently a time-consuming kind of search strategy, the more efficient ones like decision trees and k nearest neighbors (k-NN) will be better choices. For (2), theoretically, the range of *N* could be from 1 to ∞ (this makes no sense of course), but too large value of *N* will increase the complexity of a classifier ensemble system [30], while if *N* is too small, the performance of the GACEM will deteriorate without enough diverse classifiers, so the search for an appropriate *N* is a heuristic process and we will discuss it in Section 4.2. For (3), as we know, although there has been a lot of prevailing approaches such as voting and averaging [11, 31], none has been proved to be the panacea. The choice is indeed more of an art than a science. But it has been proved that ensemble *many* instead of *all* of the classifiers at hand could achieve better performance [23]. So the basic idea in GACEM is among all *N* classifiers, just taking those whose weights (i.e. *ω*) are bigger than a pre-set threshold *λ* to join the ensemble and ignoring the others. And the effect of different combination function will be discussed in Section 4.2.3.

The flowchart of GACEM is shown as below:Input:

*M*Number of sensors*N*Number of classifiers*Classifier_Bas_*Basic classifier*F_dc_*Decision combination function*λ*Threshold for classifier selection*S_train_*Training set*S_val_*Validation set*nPop*Population size*I*_max_Terminal number of generations*L_fit_*Value of fitness limit*P_c_*Crossover probability*P_m_*Mutation probability


Procedure:

Step 1.Generate initial population of chromosomes.
Step 2.Evaluate fitness (classification accuracy on *S_val_*) of each new chromosome: for *i* = 1 : *nPop* {  Decoding the *i* -th chromosome and building *N* classifiers based on *S*_train_;  Choosing those classifiers whose weight is bigger than *λ* to construct the classifier ensemble;  Calculating the classification accuracy (i.e., fitness of the *i* -th chromosome) of *S_val_* using the generated classifier ensemble;  Find the chromosome with highest fitness 
$Chm_{b}^{0}$ among the population; }
Step 3.Are the optimization criteria met? If YES, go to step 9. If NO, go to step 4.
Step 4.Generate new population using the selection operator.
Step 5.Perform the crossover operator according to the crossover probability *P_c_*.
Step 6.Perform the mutation operator according to the mutation probability *P_m_*.
Step 7.Evaluate fitness of each new chromosome: for *i* = 1 : *nPop* {  Decoding the *i* -th chromosome and building *N* classifiers based on *S_train_*;  Choosing those classifiers whose weight is bigger than *λ* to construct the classifier ensemble;  Calculating the classification accuracy (i.e., fitness of the *i* -th chromosome) of *S_val_* using the generated classifier ensemble;  Find the chromosome with highest fitness *Chm_b_* and the worst one *Chm_w_*; }
Step 8.Find the best chromosome during the evolution history and guarantee its survival to the next generation, i.e., comparing *Chm_b_* and, 
$Chm_{b}^{0}$ if the fitness of 
$Chm_{b}^{0}$ is greater than *Chm_b_*, then replace *Chm_w_* with; 
$\mathrm{Ch}m_{b}^{0}$ otherwise replace 
$Chm_{b}^{0}$ with *Chm_b_*. Go to step 3.
Step 9.End.


## 4. Experimental Section

### 4.1. Experiment description

#### 4.1.1 Experiment environment

**Figure 5.**
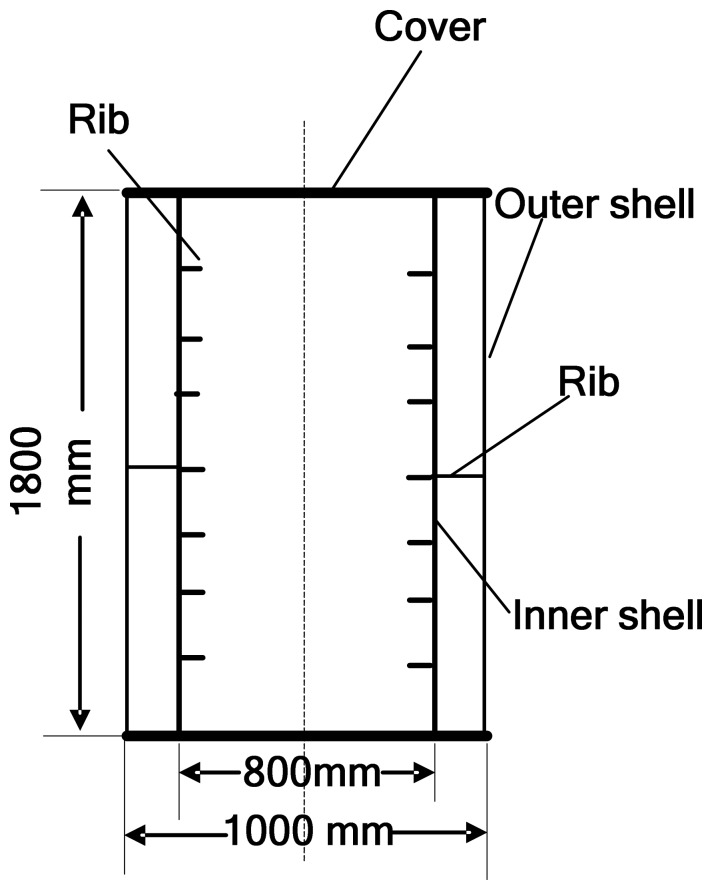
Structure of the ribbed double-shell model.

**Figure 6.**
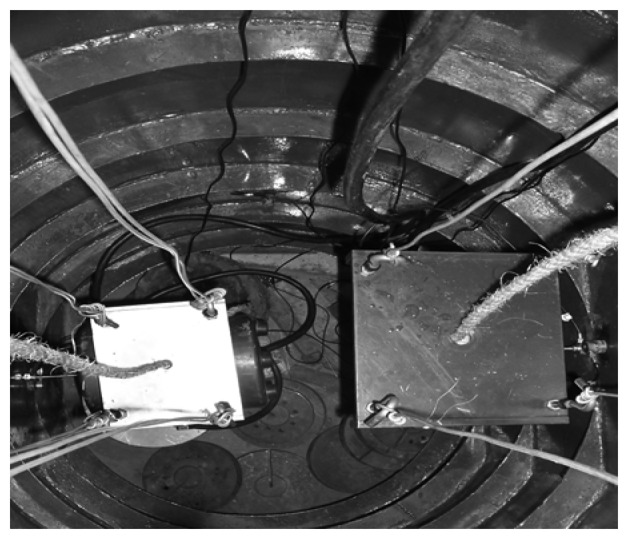
Positions of two exciters.

**Figure 7.**
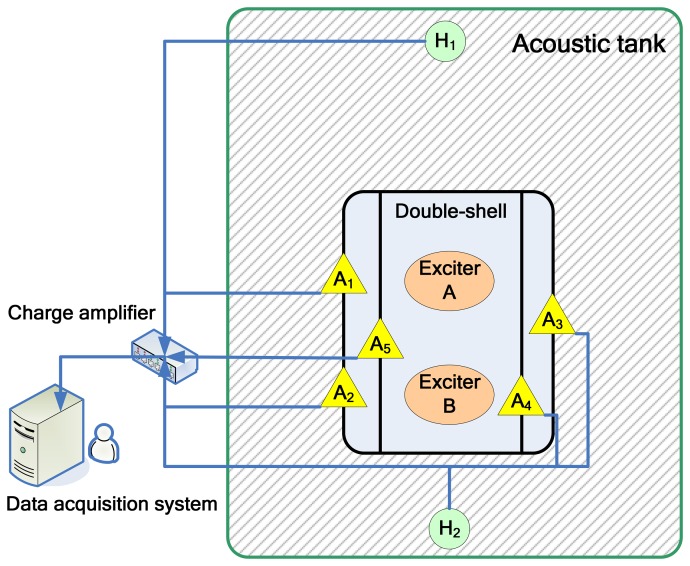
Sketch of the experiment.

There have been a number of applications of MSS in modern engineering and sound source classification is one of them. In order to acquire a better estimation of the sound source's characters, a number of sensors are used for condition monitoring and data acquisition. For example, [32] demonstrated utilization of an onboard MSS for monitoring and diagnosis of ship's acoustic health. In this article, an analogous experiment is designed. A ribbed cylindrical double-shell (see Figure 5) is built for simulation of the cabin of ship with reduced scale size and two vibration exciters (see Figure 6) are placed in the double-shell to simulate sound source by working at different frequency condition (See Table 1). Moreover, seven sensors including five accelerometers and two hydrophones are used for data acquisition in different positions (See Table 2). The overall sketch map of the experiment can be found in in Figure 7.

#### 4.1.2 Feature generation

In our experiment, the sampling frequency is 1 kHz and the analyzing frequency is 500 Hz. For each sound source, the sampling time is 10 s, so the time series of each sound source contains 10,000 points. When extracting data samples from the recordings, we choose the segments of continuous 512 points from the beginning in turn. Then the number of data samples of each sound source is 19 and among them, four are picked out for training, five for validation in the fitness function and 10 for testing the generalization. So the total number of data samples in training set, validation set and test set of all sound sources is 140, 175 and 350 respectively. And for a given sound source, the data samples in different sets are all I.I.D (Independent Identically Distributed) due to the steady signal character of the source. The detailed introduction of different sample sets can be found in Table 3.

**Figure 8.**
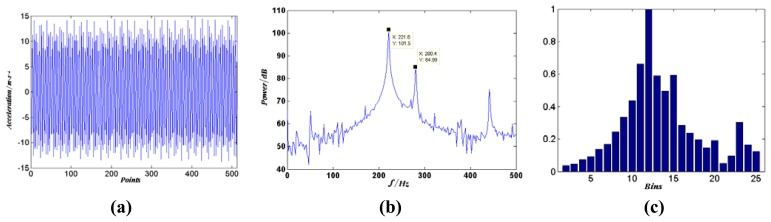
Demonstration of (a) time series and (b) power spectrum and (c) feature vector of the 22^nd^ sound source.

After computing the power spectrum of each raw data pattern, we then divide the spectrum vector from 0 to 500 Hz into 25 equal-width bins each holding 20 Hz frequency band. And the sum of each bin is taken as one dimension of the feature vector for the classification. So the raw data sample can be transformed into a 25-dimensional feature vector. Supposing *x* = [*x*_1_,…,*x*_25_] represents such a feature vector, it is then to be scaled through the following step:

(3) $$x_{i}=\frac{x_{i}-\min(\text{x})}{\max(\text{x})-\min(\text{x})},\text{i}=1,\ldots ,25$$

to ensure all the elements of *x* will vary between [0,1]. For example, the time series, power spectrum and feature vector of one sample of the 22^nd^ sound source signal in channel A_1_ are shown in Figure 8.

#### 4.1.3 Experimental methodology

In our experiments, GACEM is compared with the conventional approaches, i.e. feature-level fusion (FLF), decision-level fusion (DLF), and the single basic classifier generated on the Sensor channel with the Best Performance (SBP). Here the genetic algorithm employed by GACEM is realized in MATLAB 7.1. The experiments with GACEM are confined to four basic types of classifiers: (1) Linear Discriminant Classifier (LDC) [33], (2) Quadratic Discriminant Classifier (QDC) [33], (3) k-Nearest Neighbor (k-NN) [34] and (4) Classification And Regression Trees (CART) [35]. Besides, in one round performance comparison among FLF, DLF, SBP and GACEM, the selected basic classifiers are identical. Here we do not optimize the architecture and the parameters of those basic classifiers because we care the relative performance of the ensemble approaches instead of their absolute performance. What's more, as mentioned above, *F_DC_* can be arbitrary rule. Without the loss of generality, we adopt the plurality voting as the decision combination function.

The number of classifiers *N* and the threshold *λ* may be the most difficult input parameters to give because there is no general rule to follow. So we will discuss the influence of them on GACEM's performance with different value in the next section. The other input parameters are listed as follows: *M* = 7, *nPop* = 30, *I*_max_=100, *L_fit_*=0.99, *P_c_*=0.8, *P_m_*=0.2.

### 4.2. Results and discussion

#### 4.2.1 Performance with *N* = *M* and *λ* = 0.05

**Figure 9.**
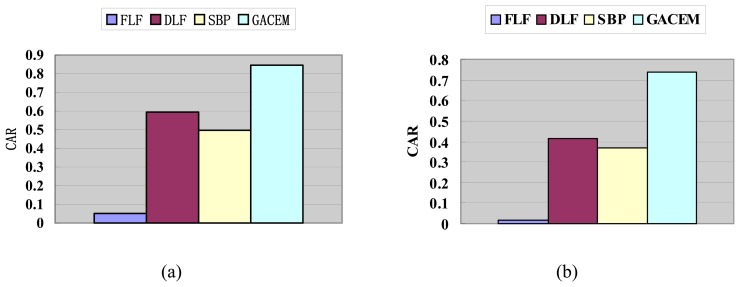

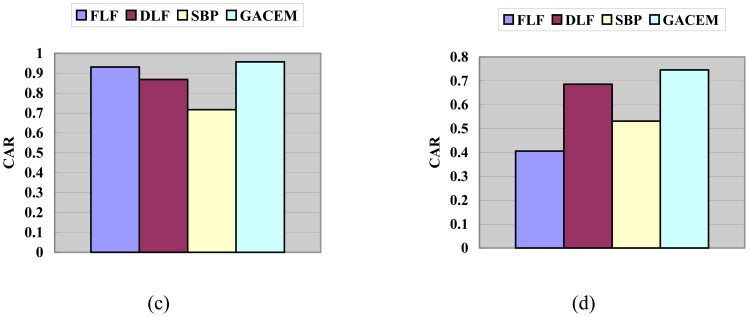
Classification accuracy rate of GACEM with different basic classifier: (a) LDC, (b) QDC, (c) k-NN and (d) CART

In this test, we assume that *N* = *M* and *λ* = 0.05. And the plurality voting is adopted as the decision combination function. The results of the Classification Accuracy Rate (CAR) of GACEM with different basic classifier are given in Figure 9.

**Figure 10.**
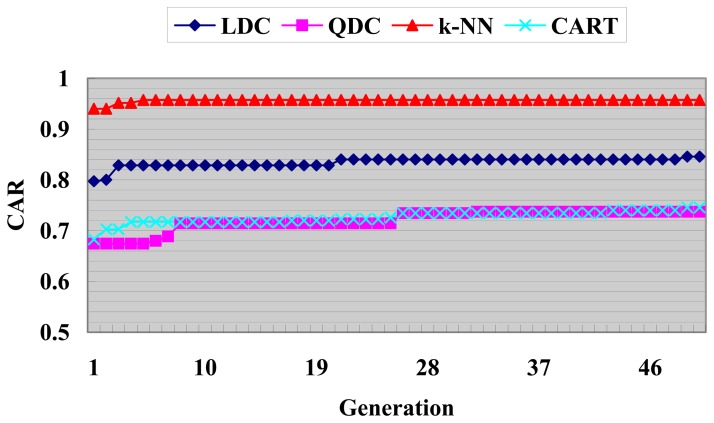
The best fitness curve versus generation.

The best fitness function value versus generation of GACEM with different basic classifier is shown below in Figure 10.

Moreover, the chromosome individual with the best fitness of GACEM has been encoded in Table 4. Each row represents the feature source of the classifier, for example, in Table 4(a), the first classifier *f*_1_ is built on feature from the 2^nd^ sensor channel (H_2_) and its weight is 0.2075. Because our given threshold is 0.05, so *f*_1_ is accepted into the classifier ensemble system.

Figure 9 shows that with any of the four kind of listed basic classifiers, i.e., LDC, QDC, k-NN and CART, GACEM yields the highest classification accuracy rate. This shows that GACEM has done the job of searching a more appropriate fusion strategy than FLF and DLF. What's more, the variance of FLF, DLF, SBP and GACEM's CAR over the four basic classifiers are 0.1804, 0.0358, 0.0204 and 0.0106 respectively. This means that GACEM is the most robust approach among them and on the contrary FLF tends to be affected the choice of basic classifier dramatically.

From the best fitness evolutionary curve shown in Figure 10, we find that the uptrend still occurs even in the last few generations except for curve of k-NN (The reason may be that k-NN's CAR has been already high enough). So if we enlarge the value of *I*_max_ with the permission of time consuming, GACEM may have the potential to achieve better performance.

Finally, it can be found that in the classifier represented in Table 4, none is discarded due to its weight. That is to say, all the classifiers available have been considered qualified for chosen into GACEM. It suggests that there is still useful information hidden in the features and more classifiers could lead to a further mining.

#### 4.2.2 Performance with *N* = 3*M* and *λ* = 1/ *N*

We then choose *N* = 3*M*, *λ* = 1/ *N* and also adopt the plurality voting as the decision combination function. A natural explanation for choosing *λ* is that the classifier whose weight is less than the average (1/ *N*) will contribute little for ensemble.

**Figure 11.**
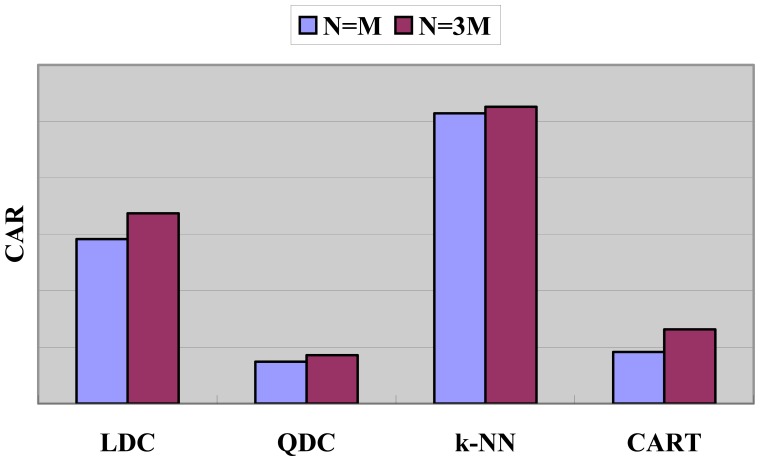
CAR of GACEM with *N* = 3*M* and *λ* = 1/ *N*.

Comparison of CAR when *N* = 3*M* and *N* = *M* is demonstrated in Figure 11. We find that CAR does have been improved on all kinds of basic classifier, which proves that our hypothesis of enlarging the value of *N* is helpful.

**Figure 12.**
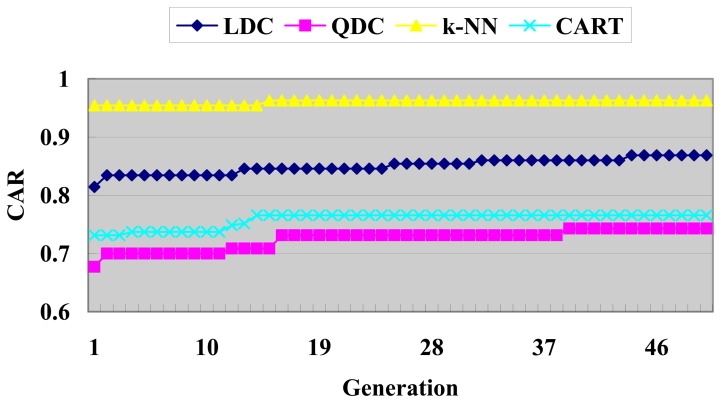
The best fitness curve versus generation.

Also, the best fitness function value versus generation of GACEM with different basic classifier is shown below in Figure 12. Like Figure 10, it is further believed that more generations will yield better performance because of the existence of uptrend in the last few generations.

Surprisingly, when *N* = 3*M*, the number of selected classifiers in ensemble is 7, 11, 3 and 12 using LDC, QDC, k-NN and CART respectively. In particular, when the basic classifier is k-NN, over all 21 (*N* = 3*M* = 21) generated classifiers, only three of them are chosen for ensemble (see Table 5). On the contrary, the performance is even better than the ensemble consisting of seven classifiers presented in Table 4(c). This means that GACEM can generate classifier ensembles with far smaller sizes but more powerful classification ability.

#### 4.2.3 Performance comparison among different combination functions

Another important factor in classifier ensemble is the combination function. In this section, majority voting, plurality voting and weighted averaging are selected in GACEM respectively. Here, we set the weight of each classifier in the chromosome as its weight when averaging.

**Figure 13.**
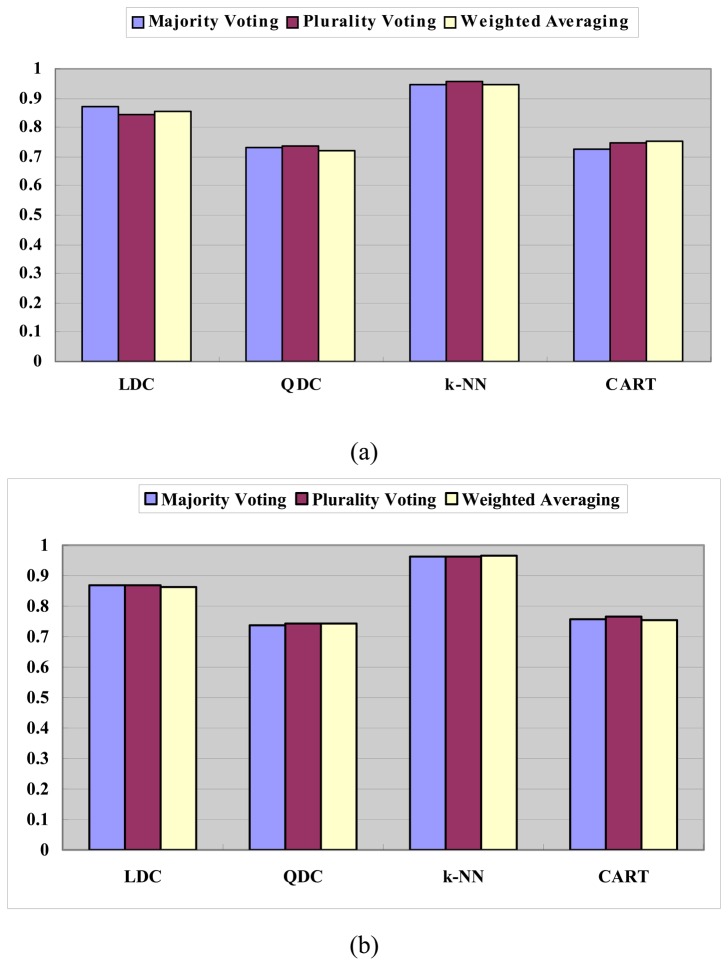
Classification accuracy rate of GACEM with different combination functions: (a) *N* = *M*, *λ* = 0.05 and (b) *N* = 3*M*, *λ* = 1/ *N*.

When *N* = *M*, *λ* = 0.05 and the other parameters are the same as in Section 4.2.1. The results of experiments are given in Figure 13(a). When *N* = 3*M*, *λ* = 1/ *N* and the other parameters are the same as in Section 4.2.2. The results of experiments are given in Figure 13(b).

Figure 13 shows: fixing the basic classifier, the CAR of GACEM varies little among the three kind of listed combination functions, i.e., majority voting, plurality voting and weighted averaging. This means that GACEM is not so sensitive to the selection of combination function.

## 5. Conclusions

The experimental study shows that GACEM is superior to both the conventional feature-level fusion and decision-level fusion because it utilizes the combination of more than one classifier to obtain a more precise classification result. Besides, GACEM is able to choose the *elites* for ensemble among the classifiers where the good and bad are intermingled, which could reduce the complexity of the classifier ensemble system remarkably.

Note that although GACEM has obtained impressive performance in our empirical study, we believe that there are still some candidate improvement directions on GACEM: (1) taking more sophisticated and powerful classifier such as support vector machine (SVM) as the basic classifier, (2) improving the basic classifiers by synergizing with subsampling the training examples such as Bagging or Boosting and (3) using different basic classifier for different subset of features set by adding extra gene positions to indicate both the basic classifier's type and parameters and then allowing the GA to search the optimal setting. Also, it is feasible to design algorithms for sensor selection [36, 37] along the way that GACEM goes.

## Figures and Tables

**Table 1.** List of 35 kinds of sound sources.

**Sound source ID**	1	2	3	4	5	6	7	8	9	10
***f****_A_***(Hz)**	0	0	0	0	0	20	20	20	20	20
***f****_B_***(Hz)**	20	110	220	280	320	0	20	110	220	280

**Sound source ID**	11	12	13	14	15	16	17	18	19	20

***f****_A_***(Hz)**	20	110	110	110	110	110	110	220	220	220
***f****_B_***(Hz)**	320	0	20	110	220	280	320	0	20	110

**Sound source ID**	21	22	23	24	25	26	27	28	29	30

***f****_A_***(Hz)**	220	220	220	280	280	280	280	280	280	320
***f****_B_***(Hz)**	220	280	320	0	20	110	220	280	320	0

**Sound source ID**	31	32	33	34	35					

***f****_A_***(Hz)**	320	320	320	320	320					
***f****_B_***(Hz)**	20	110	220	280	320					

Note:
● There are 35 kinds of different sound sources in all. ● *f_A_* represents the working frequency of exciter A and *f_B_* represents the working frequency of exciter B. ● 0 Hz means the exciter is unused.

**Table 2.** Description of sensors.

**Sensor NO.**	**Sensor Type (ID)**	**Position**
1	Hydrophone (H_1_)	Far field
2	Hydrophone (H_2_)	Near field
3	Accelerometer (A_1_)	Outer shell
4	Accelerometer (A_2_)	Outer shell
5	Accelerometer (A_3_)	Outer shell
6	Accelerometer (A_4_)	Inner shell
7	Accelerometer (A_5_)	Inner shell

**Table 3.** Detailed aggregation of training set, validation set and test set.

**Sound source ID**	1	2	3	4	5	6	7	8	9	10
**Training set**	4	4	4	4	4	4	4	4	4	4

**Validation set**	5	5	5	5	5	5	5	5	5	5
**Test set**	10	10	10	10	10	10	10	10	10	10

**Sound source ID**	11	12	13	14	15	16	17	18	19	20

**Training set**	4	4	4	4	4	4	4	4	4	4

**Validation set**	5	5	5	5	5	5	5	5	5	5
**Test set**	10	10	10	10	10	10	10	10	10	10

**Sound source ID**	21	22	23	24	25	26	27	28	29	30

**Training set**	4	4	4	4	4	4	4	4	4	4

**Validation set**	5	5	5	5	5	5	5	5	5	5
**Test set**	10	10	10	10	10	10	10	10	10	10

**Sound source ID**	31	32	33	34	35	Total				

**Training set**	4	4	4	4	4	140				

**Validation set**	5	5	5	5	5	175				
**Test set**	10	10	10	10	10	350				

**Table 4.** Encoded chromosome individual with the best fitness on different basic classifier: (a) LDC, (b) QDC, (c) k-NN and (d) CART.

		
	H_1_	H_2_	A_1_	A_2_	A_3_	A_4_	A_5_	Weight			H_1_	H_2_	A_1_	A_2_	A_3_	A_4_	A_5_	Weight
		
*f*_1_	0	1	0	0	0	0	0	0.2075		*f*_1_	0	0	1	1	0	1	1	0.2068
*f*_2_	0	0	0	1	1	1	0	0.0521		*f*_2_	0	1	0	1	0	1	1	0.2214
*f*_3_	0	0	1	0	1	1	0	0.1354		*f*_3_	0	0	0	1	1	0	1	0.1713
*f*_4_	0	0	1	1	0	0	1	0.1781		*f*_4_	0	1	1	0	1	0	1	0.086
*f*_5_	1	0	0	0	0	0	1	0.0688		*f*_5_	0	1	1	0	1	1	1	0.096
*f*_6_	0	0	0	0	0	1	1	0.1634		*f*_6_	1	0	0	0	1	0	0	0.1436
*f*_7_	0	0	1	1	1	0	1	0.1948		*f*_7_	0	1	1	1	1	0	0	0.0749
		
(a)										(b)								

		
	H_1_	H_2_	A_1_	A_2_	A_3_	A_4_	A_5_	Weight			H_1_	H_2_	A_1_	A_2_	A_3_	A_4_	A_5_	Weight
		
*f*_1_	0	0	1	1	0	0	0	0.1367		*f*_1_	1	1	0	0	1	0	1	0.2213
*f*_2_	0	1	0	1	0	1	1	0.0578		*f*_2_	1	1	1	0	0	1	0	0.1335
*f*_3_	0	1	0	1	1	0	0	0.2177		*f*_3_	0	0	0	0	1	0	1	0.1374
*f*_4_	0	1	0	0	0	1	1	0.1225		*f*_4_	0	0	1	0	0	1	0	0.1258
*f*_5_	1	0	1	0	1	0	1	0.1507		*f*_5_	0	0	1	0	0	1	0	0.1976
*f*_6_	1	1	1	1	1	1	1	0.1777		*f*_6_	0	1	0	1	1	0	1	0.0821
*f*_7_	1	1	0	1	0	0	1	0.1369		*f*_7_	0	0	0	1	0	1	1	0.1024
		
(c)										(d)								

**Table 5.** Encoded chromosome individual with the best fitness on k-NN, noting that only *f*_15_, *f*_16_, and *f*_19_ whose weight is greater than the threshold (*λ* =1/ *N* ≈ 0.047) are selected for ensemble.

	H_1_	H_2_	A_1_	A_2_	A_3_	A_4_	A_5_	Weight
*f*_1_	1	1	1	0	0	1	1	0.0280
*f*_2_	0	0	0	1	0	1	1	0.0109
*f*_3_	0	1	0	1	0	0	1	0.0095
*f*_4_	1	1	1	1	1	1	1	0.0110
*f*_5_	0	1	1	1	0	0	1	0.0261
*f*_6_	0	0	1	0	1	1	0	0.0068
*f*_7_	0	1	0	1	1	1	0	0.0082
*f*_8_	1	0	1	1	0	1	1	0.0083
*f*_9_	1	0	1	1	0	1	1	0.0125
*f*_10_	1	0	0	1	1	0	0	0.0091
*f*_11_	1	1	0	1	0	1	0	0.0277
*f*_12_	1	1	0	0	1	0	1	0.0184
*f*_13_	0	1	1	0	1	0	1	0.0049
*f*_14_	1	1	0	0	0	1	0	0.0113
*f*_15_	***0***	***1***	***0***	***1***	***0***	***1***	***1***	***0.1960***
*f*_16_	***0***	***0***	***1***	***1***	***0***	***0***	***0***	***0.4410***
*f*_17_	0	1	1	1	1	0	1	0.0132
*f*_18_	1	1	0	1	0	0	0	0.0053
*f*_19_	***1***	***1***	***0***	***1***	***1***	***1***	***0***	***0.0964***
*f*_20_	0	0	1	1	1	1	0	0.0186
*f*_21_	0	0	1	1	1	1	1	0.0359

## References

[1] Rajagopal R., Sankaranarayanan B., Rao P R. (1990). Target Classification in A Passive Sonar - An Expert System Approach. International Conference on Acoustics, Speech, and Signal Processing.

[2] Smith D., Singh S. (2006). Approaches to Multisensor Data Fusion in Target Tracking: A Survey. IEEE Transactions on Knowledge and Data Engineering.

[3] Kittler J., Matas J., Jonsson K., Ramos Sanchez M. U. (1997). Combining Evidence in Personal Identity Verification Systems. Pattern Recognition Letters.

[4] Kacalenga R., Erickson D., Palmer D. (2003). Voting Fusion for Landmine Detection. IEEE Aerospace and Electronic Systems Magazine.

[5] Costa A. D., Sayeed A.M. (2003). Data versus Decision Fusion in Wireless Sensor Networks. International Conference on Acoustics, Speech, and Signal Processing.

[6] Brooks R.R., Ramanathan P., Sayeed A.M. (2003). Distributed Target Classification and Tracking in Sensor Networks. Proceedings of the IEEE.

[7] Luo R.C., Yih C.-C., Su K. L. (2002). Multisensor Fusion and Integration: Approaches, Applications, and Future Research Directions. IEEE Sensors Journal.

[8] Hall D.L., Llinas J. (1997). An Introduction to Multisensor Data Fusion. Proceedings of the IEEE.

[9] Clouqueur T., Ramanathan P., Saluja K. K., Wang K.-C. (2001). Value-Fusion versus Decision-Fusion for Fault-Tolerance in Collaborative Target Detection in Sensor Networks. Proc. 4th Ann. Conf. on Information Fusion.

[10] Roli F., Giacinto G., Vernazza G. (2001). Methods for Designing Multiple Classifier Systems. Proceedings of the Second International Workshop on Multiple Classifier Systems.

[11] Kittler J., Hatef M., Duin R.P.W., Matas J. (1998). On Combining Classifiers. IEEE Transactions on Pattern Analysis and Machine Intelligence.

[12] Kuncheva L.I., Whitaker C.J. (2003). Measures of Diversity in Classifier Ensembles and Their Relationship with the Ensemble Accuracy. Machine Learning.

[13] Hansen L.K., Salamon P. (1990). Neural Network Ensembles. IEEE Transactions on Pattern Analysis and Machine Intelligence.

[14] Ueda N. (2000). Optimal Linear Combination of Neural Networks for Improving Classification Performance. IEEE Transactions on Pattern Analysis and Machine Intelligence.

[15] Kittler J. (2001). Multi-Sensor Integration and Decision Level Fusion. Proc. DERA/IEE Workshop Intelligent Sensor Processing.

[16] Polikar R., Parikh D., Shreekanth Mandayam. (2006). Multiple Classifier Systems for Multisensor Data Fusion. SAS 2006 - IEEE Sensors Applications Symposium.

[17] Schapire R. E. (1999). A Brief Introduction to Boosting. Proceedings of the Sixteenth International Joint Conference on Artificial Intelligence.

[18] Dietterich T.G. (1997). Machine learning research: Four current directions. AI Magazine.

[19] Breiman L. (1996). Bagging Predictors. Machine Learning.

[20] Xu R., He L., Zhang L., Ben K. (2008). Identification of Mechanical Noise Source on Sparse Data. Chinese Journal of Mechanical Engineering.

[21] Roli F., Giacinto G., Serpico S.B. (2001). Classifier Fusion for Multisensor Image Recognition. Proceedings of SPIE - Image and Signal Processing for Remote Sensing.

[22] Kuncheva L.I., Jain L. C. (2000). Designing Classifier Fusion Systems by Genetic Algorithms. IEEE Transactions on Evolutionary Computation.

[23] Zhou Z.-H., Wu J., Tang W. (2002). Ensembling Neural Networks: Many Could Be Better Than All. Artificial Intelligence.

[24] Tumer K., Ghosh J., Sharkey A. (1999). Linear and Order Statistics Combiners for Pattern Classification. Combining Artificial Neural Nets: Ensemble and Modular Multi-Net Systems.

[25] Lam L., Suen C.Y. (1997). Application of majority voting to pattern recognition: An analysis of the behavior and performance. IEEE Transactions on Systems, Man, and Cybernetics.

[26] Lin X., Yacoub S., Burns J., Simske S. (2003). Performance analysis of pattern classifier combination by plurality voting. Pattern Recognition Letters.

[27] Perrone M. P. (1993). Improving Regression Estimation: Averaging Methods for Variance Reduction with Extensions to General Convex Measure Optimization.

[28] Maslov I.V., Gertner I. (2006). Multi-sensor fusion: an Evolutionary algorithm approach. Information Fusion.

[29] Buczak A.L., Uhrig R.E. (1996). Hybrid Fuzzy-Genetic Technique for Multisensor Fusion. Information Sciences.

[30] Ruta D., Gabrys B. (2005). Classifier Selection for Majority Voting. Information Fusion.

[31] Narasimhamurthy A. (2005). Theoretical Bounds of Majority Voting Performance for a Binary Classification Problem. IEEE Transactions on Pattern Analysis and Machine Intelligence.

[32] Seto M. L., Hutt D. (2004). Ship Signatures Management System – Towards increased warship survivability. Underwater Defence Technology.

[33] Friedman J.H. (1989). Regularized Discriminant Analysis. Journal of the American Statistical Association.

[34] Cover T., Hart P. (1967). Nearest Neighbor Pattern Classification. EEE Transactions on Information Theory.

[35] Lawrence R.L., Wright A. (2001). Rule-based Classification Systems Using Classification And Regression Tree (CART). Analysis Photogrammetric Engineering & Remote Sensing.

[36] Gardner J.W., Boilot P., Hines E.L. (2005). Enhancing Electronic Nose Performance by Sensor Selection Using a New Integer-based Genetic Algorithm Approach. Sensors and Actuators B.

[37] Worden K., Burrows A.P. (2001). Optimal Sensor Placement for Fault Detection. Engineering Structures.

